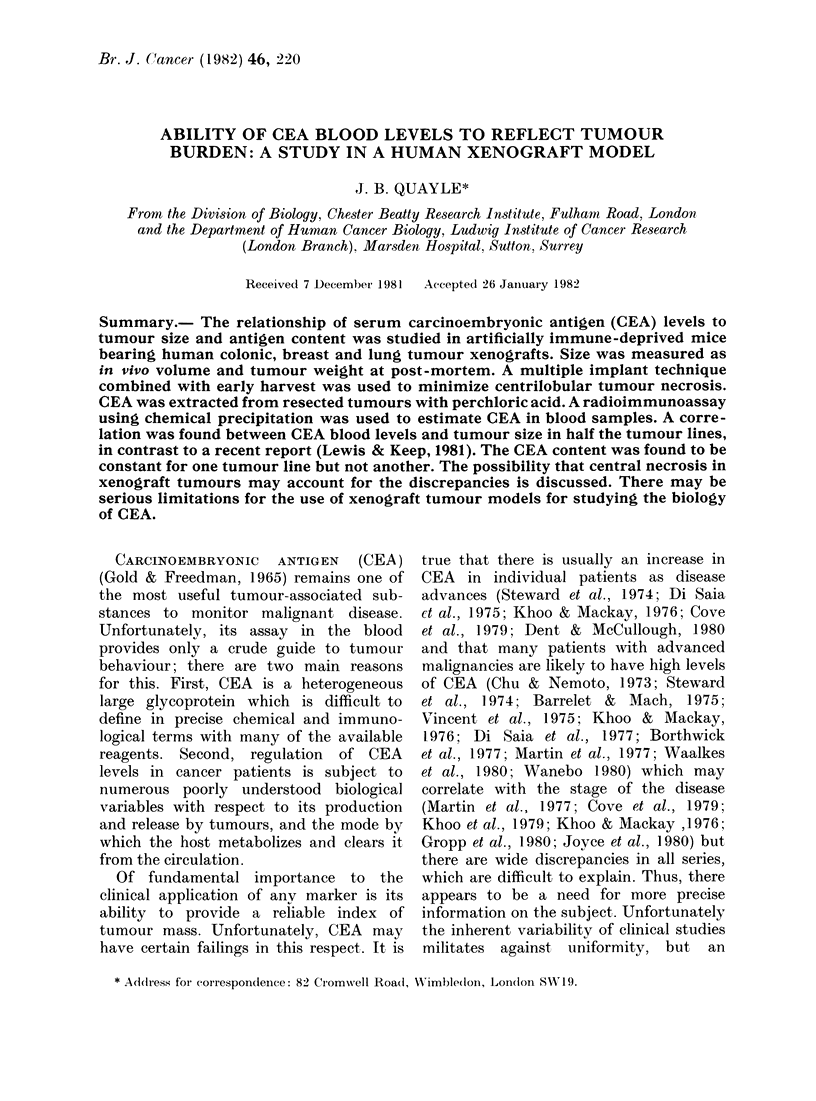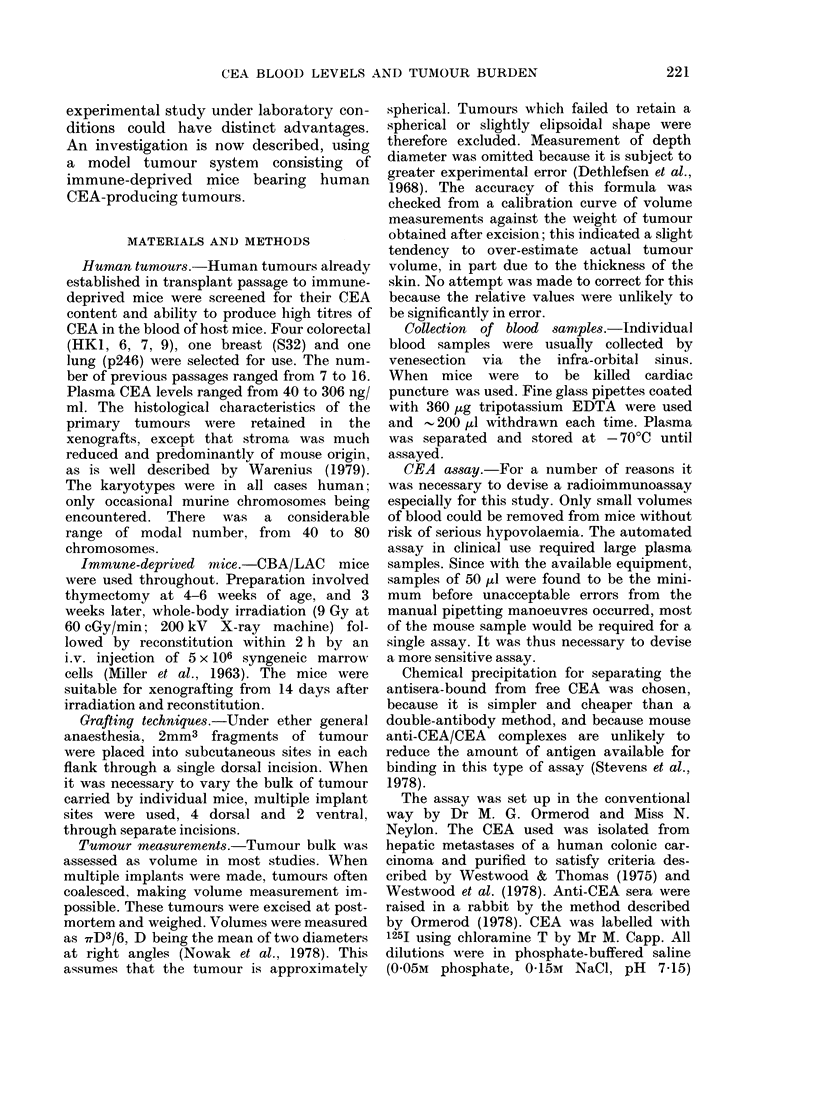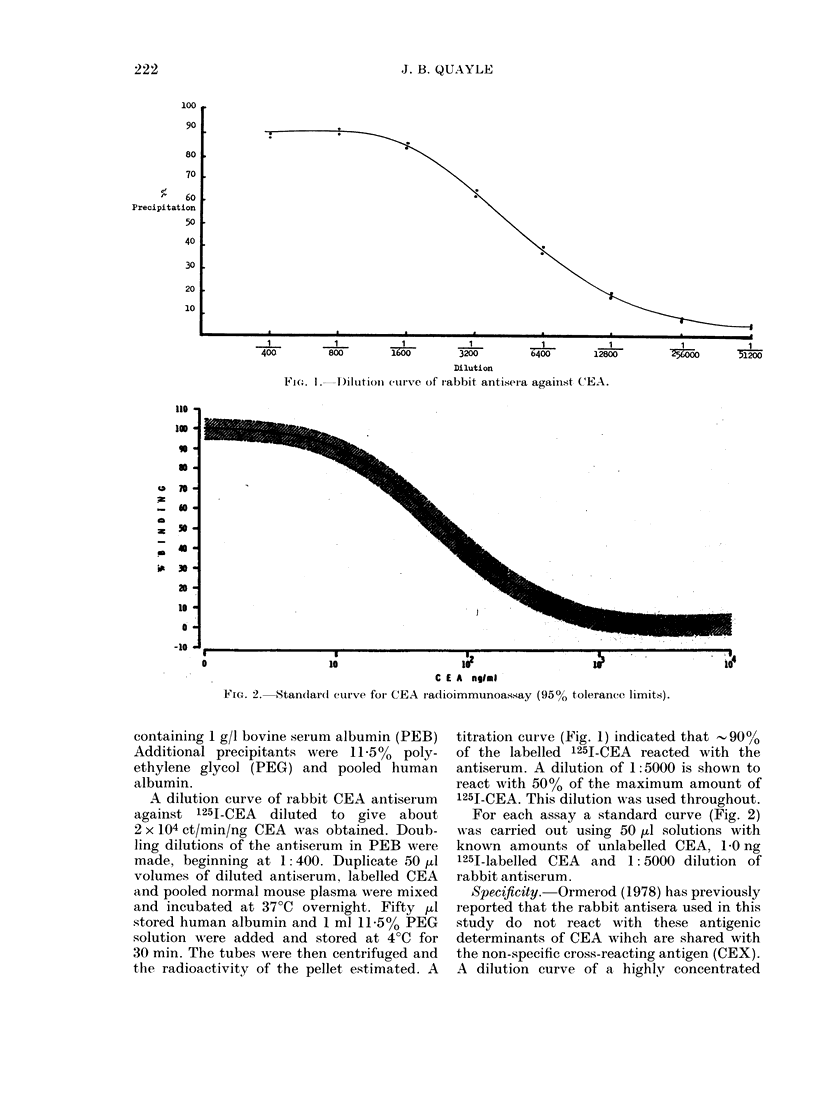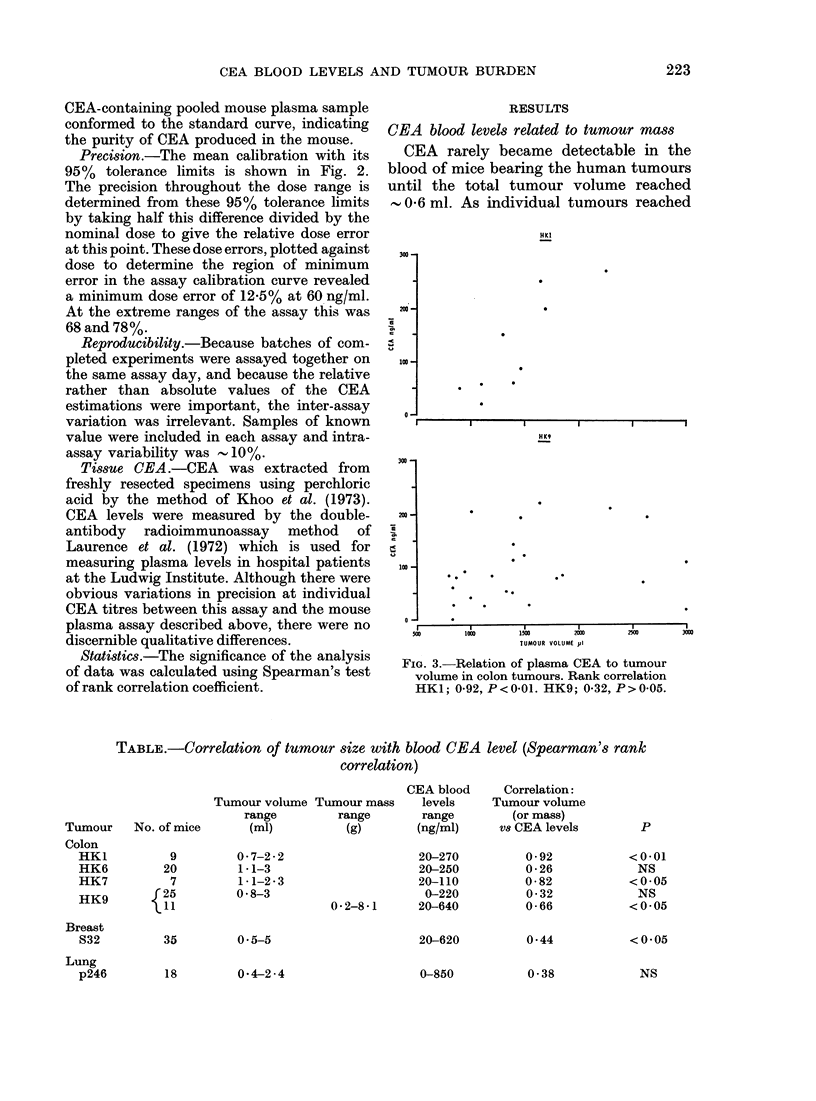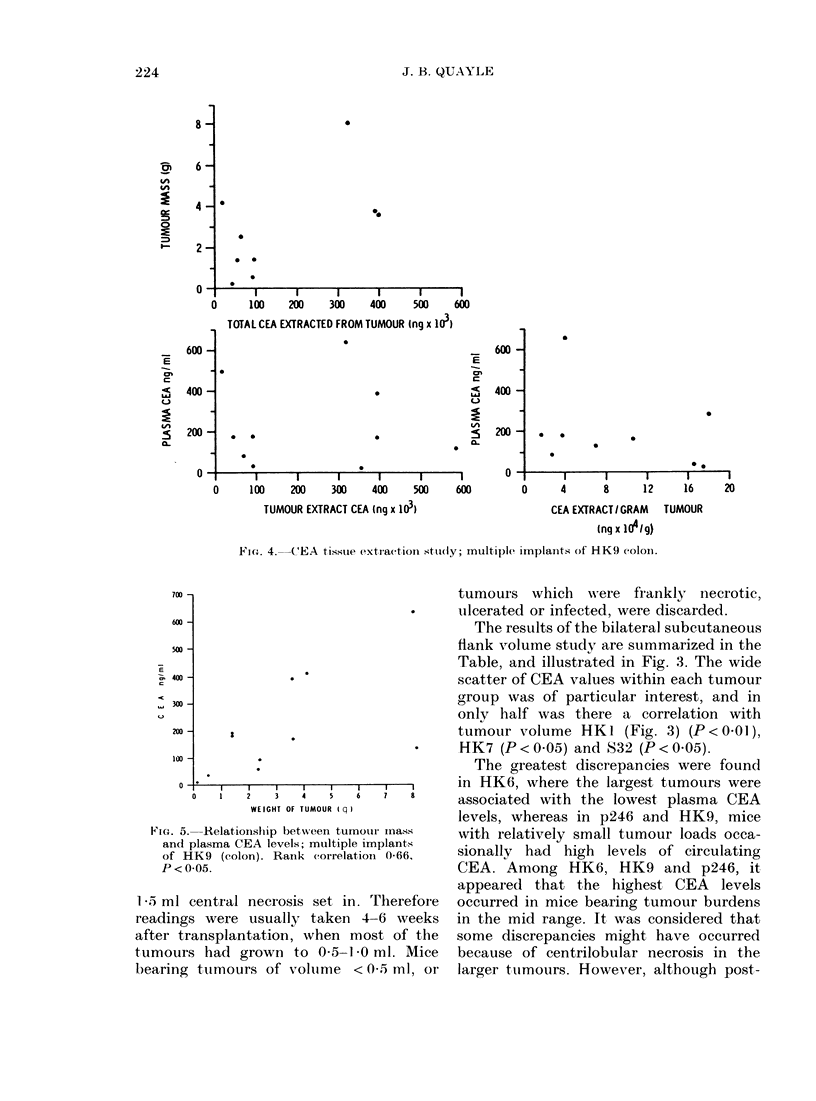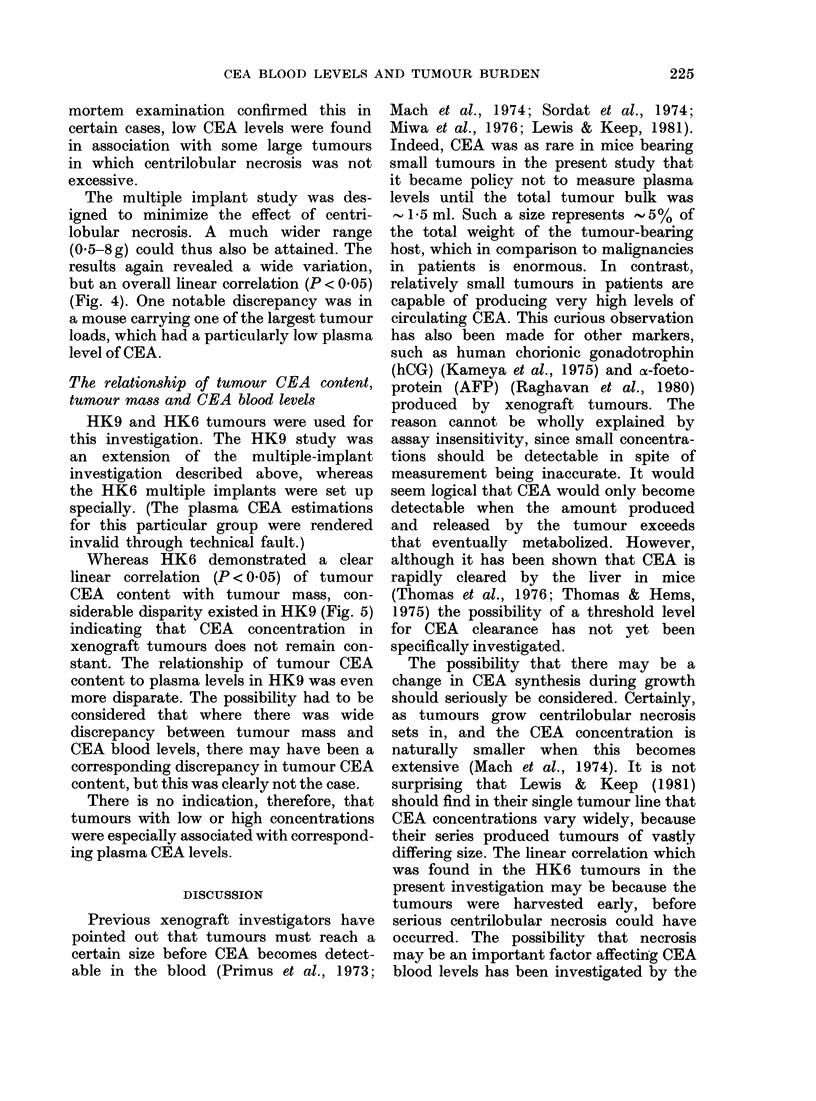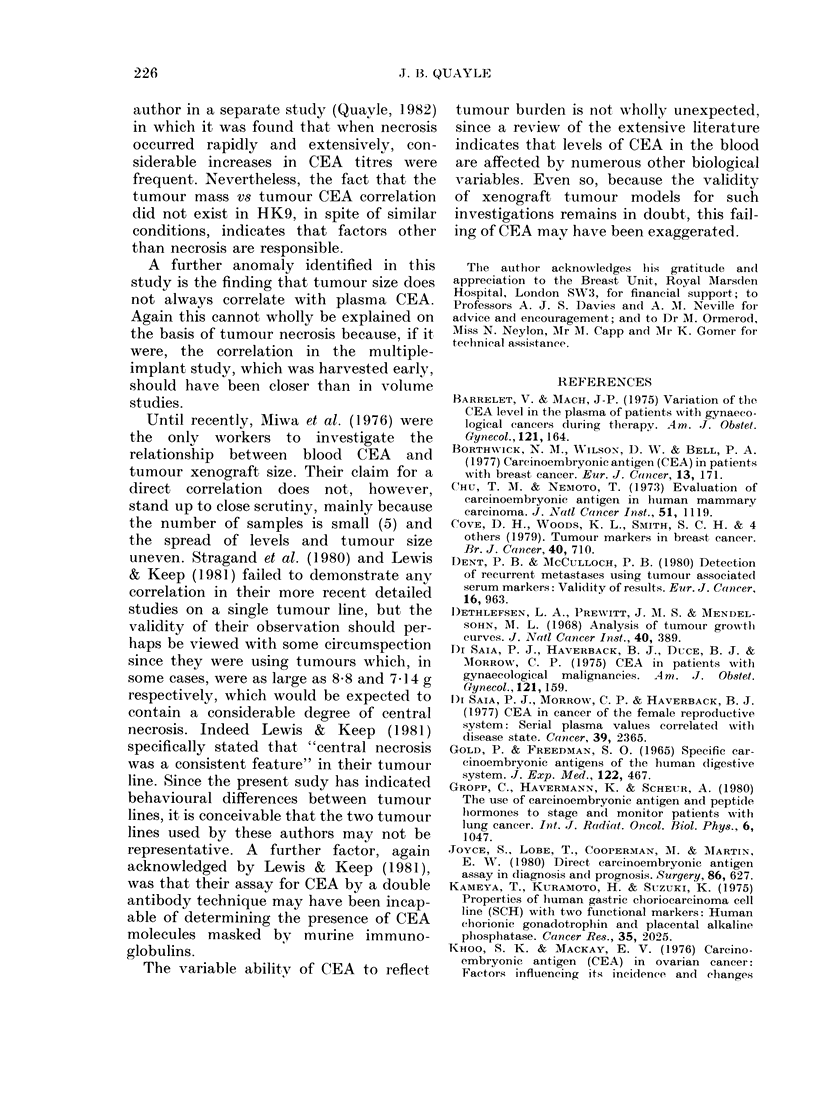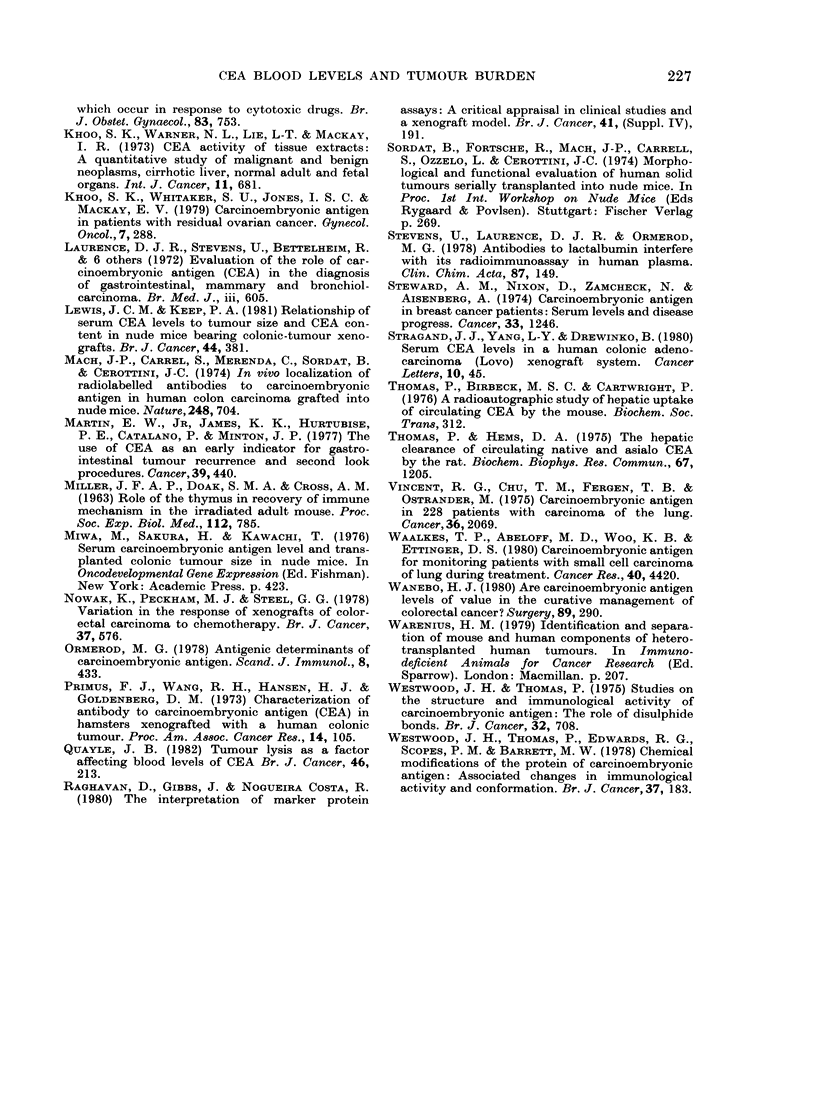# Ability of CEA blood levels to reflect tumour burden: a study in a human xenograft model.

**DOI:** 10.1038/bjc.1982.187

**Published:** 1982-08

**Authors:** J. B. Quayle

## Abstract

The relationship of serum carcinoembryonic antigen (CEA) levels to tumour size and antigen content was studied in artificially immune-deprived mice bearing human colonic, breast and lung tumour xenografts. Size was measured as in vivo volume and tumour weight at post-mortem. A multiple implant technique combined with early harvest was used to minimize centrilobular tumour necrosis. CEA was extracted from resected tumours with perchloric acid. A radioimmunoassay using chemical precipitation was used to estimate CEA in blood samples. A correlation was found between CEA blood levels and tumour size in half the tumour lines, in contrast to a recent report (Lewis & Keep, 1981). The CEA content was found to be constant for one tumour line but not another. The possibility that central necrosis in xenograft tumours may account for the discrepancies is discussed. There may be serious limitations for the use of xenograft tumour models for studying the biology of CEA.


*Br. J. Cancer* (1982) **46**, 220

### ABILITY OF CEA BLOOD LEVELS TO REFLECT TUMOUR BURDEN: A STUDY IN A HUMAN XENOGRAFT MODEL

J. B. QUAYLE*

*From the Division of Biology, Chester Beatty Research Institute, Fulham Road, London and the Department of Human Cancer Biology, Ludwig Institute of Cancer Research (London Branch), Marsden Hospital, Sutton, Surrey*

Received 7 December 1981 Accepted 26 January 1982

**Summary.— The relationship of serum carcinoembryonic antigen (CEA) levels to tumour size and antigen content was studied in artificially immune-deprived mice bearing human colonic, breast and lung tumour xenografts. Size was measured as *in vivo* volume and tumour weight at post-mortem. A multiple implant technique combined with early harvest was used to minimize centrilobular tumour necrosis. CEA was extracted from resected tumours with perchloric acid. A radioimmunoassay using chemical precipitation was used to estimate CEA in blood samples. A correlation was found between CEA blood levels and tumour size in half the tumour lines, in contrast to a recent report (Lewis & Keep, 1981). The CEA content was found to be constant for one tumour line but not another. The possibility that central necrosis in xenograft tumours may account for the discrepancies is discussed. There may be serious limitations for the use of xenograft tumour models for studying the biology of CEA.**

Carcinoembryonic antigen (CEA) (Gold & Freedman, 1965) remains one of the most useful tumour-associated substances to monitor malignant disease. Unfortunately, its assay in the blood provides only a crude guide to tumour behaviour; there are two main reasons for this. First, CEA is a heterogeneous large glycoprotein which is difficult to define in precise chemical and immunological terms with many of the available reagents. Second, regulation of CEA levels in cancer patients is subject to numerous poorly understood biological variables with respect to its production and release by tumours, and the mode by which the host metabolizes and clears it from the circulation.

Of fundamental importance to the clinical application of any marker is its ability to provide a reliable index of tumour mass. Unfortunately, CEA may have certain failings in this respect. It is true that there is usually an increase in CEA in individual patients as disease advances (Steward *et al.*, 1974; Di Saia *et al.*, 1975; Khoo & Mackay, 1976; Cove *et al.*, 1979; Dent & McCullough, 1980 and that many patients with advanced malignancies are likely to have high levels of CEA (Chu & Nemoto, 1973; Steward *et al.*, 1974; Barrelet & Mach, 1975; Vincent *et al.*, 1975; Khoo & Mackay, 1976; Di Saia *et al.*, 1977; Borthwick *et al.*, 1977; Martin *et al.*, 1977; Waalkes *et al.*, 1980; Wanebo 1980) which may correlate with the stage of the disease (Martin *et al.*, 1977; Cove *et al.*, 1979; Khoo *et al.*, 1979; Khoo & Mackay ,1976; Gropp *et al.*, 1980; Joyce *et al.*, 1980) but there are wide discrepancies in all series, which are difficult to explain. Thus, there appears to be a need for more precise information on the subject. Unfortunately the inherent variability of clinical studies militates against uniformity, but an

* Address for correspondence: 82 Cromwell Road, Wimbledon, London SW19.

experimental study under laboratory conditions could have distinct advantages. An investigation is now described, using a model tumour system consisting of immune-deprived mice bearing human CEA-producing tumours.

### MATERIALS AND METHODS

*Human tumours.*—Human tumours already established in transplant passage to immune-deprived mice were screened for their CEA content and ability to produce high titres of CEA in the blood of host mice. Four colorectal (HK1, 6, 7, 9), one breast (S32) and one lung (p246) were selected for use. The number of previous passages ranged from 7 to 16. Plasma CEA levels ranged from 40 to 306 ng/ml. The histological characteristics of the primary tumours were retained in the xenografts, except that stroma was much reduced and predominantly of mouse origin, as is well described by Warenius (1979). The karyotypes were in all cases human; only occasional murine chromosomes being encountered. There was a considerable range of modal number, from 40 to 80 chromosomes.

*Immune-deprived mice.*—CBA/LAC mice were used throughout. Preparation involved thymectomy at 4–6 weeks of age, and 3 weeks later, whole-body irradiation (9 Gy at 60 cGy/min; 200 kV X-ray machine) followed by reconstitution within 2 h by an i.v. injection of $5 \times 10^6$ syngeneic marrow cells (Miller *et al.*, 1963). The mice were suitable for xenografting from 14 days after irradiation and reconstitution.

*Grafting techniques.*—Under ether general anaesthesia, 2mm$^3$ fragments of tumour were placed into subcutaneous sites in each flank through a single dorsal incision. When it was necessary to vary the bulk of tumour carried by individual mice, multiple implant sites were used, 4 dorsal and 2 ventral, through separate incisions.

*Tumour measurements.*—Tumour bulk was assessed as volume in most studies. When multiple implants were made, tumours often coalesced, making volume measurement impossible. These tumours were excised at postmortem and weighed. Volumes were measured as $\pi D^3/6$, D being the mean of two diameters at right angles (Nowak *et al.*, 1978). This assumes that the tumour is approximately spherical. Tumours which failed to retain a spherical or slightly elipsoidal shape were therefore excluded. Measurement of depth diameter was omitted because it is subject to greater experimental error (Dethlefsen *et al.*, 1968). The accuracy of this formula was checked from a calibration curve of volume measurements against the weight of tumour obtained after excision; this indicated a slight tendency to over-estimate actual tumour volume, in part due to the thickness of the skin. No attempt was made to correct for this because the relative values were unlikely to be significantly in error.

*Collection of blood samples.*—Individual blood samples were usually collected by venesection via the infra-orbital sinus. When mice were to be killed cardiac puncture was used. Fine glass pipettes coated with 360 $\mu$g tripotassium EDTA were used and $\sim 200$ $\mu$l withdrawn each time. Plasma was separated and stored at $-70°$C until assayed.

*CEA assay.*—For a number of reasons it was necessary to devise a radioimmunoassay especially for this study. Only small volumes of blood could be removed from mice without risk of serious hypovolaemia. The automated assay in clinical use required large plasma samples. Since with the available equipment, samples of 50 $\mu$l were found to be the minimum before unacceptable errors from the manual pipetting manoeuvres occurred, most of the mouse sample would be required for a single assay. It was thus necessary to devise a more sensitive assay.

Chemical precipitation for separating the antisera-bound from free CEA was chosen, because it is simpler and cheaper than a double-antibody method, and because mouse anti-CEA/CEA complexes are unlikely to reduce the amount of antigen available for binding in this type of assay (Stevens *et al.*, 1978).

The assay was set up in the conventional way by Dr M. G. Ormerod and Miss N. Neylon. The CEA used was isolated from hepatic metastases of a human colonic carcinoma and purified to satisfy criteria described by Westwood & Thomas (1975) and Westwood *et al.* (1978). Anti-CEA sera were raised in a rabbit by the method described by Ormerod (1978). CEA was labelled with $^{125}$I using chloramine T by Mr M. Capp. All dilutions were in phosphate-buffered saline (0·05M phosphate, 0·15M NaCl, pH 7·15)

FIG. 1.—Dilution curve of rabbit antisera against CEA.

FIG. 2.—Standard curve for CEA radioimmunoassay (95% tolerance limits).

containing 1 g/l bovine serum albumin (PEB) Additional precipitants were 11·5% polyethylene glycol (PEG) and pooled human albumin.

A dilution curve of rabbit CEA antiserum against $^{125}$I-CEA diluted to give about $2 \times 10^4$ ct/min/ng CEA was obtained. Doubling dilutions of the antiserum in PEB were made, beginning at 1:400. Duplicate 50 $\mu$l volumes of diluted antiserum, labelled CEA and pooled normal mouse plasma were mixed and incubated at 37°C overnight. Fifty $\mu$l stored human albumin and 1 ml 11·5% PEG solution were added and stored at 4°C for 30 min. The tubes were then centrifuged and the radioactivity of the pellet estimated. A titration curve (Fig. 1) indicated that ~90% of the labelled $^{125}$I-CEA reacted with the antiserum. A dilution of 1:5000 is shown to react with 50% of the maximum amount of $^{125}$I-CEA. This dilution was used throughout.

For each assay a standard curve (Fig. 2) was carried out using 50 $\mu$l solutions with known amounts of unlabelled CEA, 1·0 ng $^{125}$I-labelled CEA and 1:5000 dilution of rabbit antiserum.

*Specificity.*—Ormerod (1978) has previously reported that the rabbit antisera used in this study do not react with these antigenic determinants of CEA wihch are shared with the non-specific cross-reacting antigen (CEX). A dilution curve of a highly concentrated

CEA-containing pooled mouse plasma sample conformed to the standard curve, indicating the purity of CEA produced in the mouse.

*Precision.*—The mean calibration with its 95% tolerance limits is shown in Fig. 2. The precision throughout the dose range is determined from these 95% tolerance limits by taking half this difference divided by the nominal dose to give the relative dose error at this point. These dose errors, plotted against dose to determine the region of minimum error in the assay calibration curve revealed a minimum dose error of 12·5% at 60 ng/ml. At the extreme ranges of the assay this was 68 and 78%.

*Reproducibility.*—Because batches of completed experiments were assayed together on the same assay day, and because the relative rather than absolute values of the CEA estimations were important, the inter-assay variation was irrelevant. Samples of known value were included in each assay and intra-assay variability was ~10%.

*Tissue CEA.*—CEA was extracted from freshly resected specimens using perchloric acid by the method of Khoo *et al.* (1973). CEA levels were measured by the double-antibody radioimmunoassay method of Laurence *et al.* (1972) which is used for measuring plasma levels in hospital patients at the Ludwig Institute. Although there were obvious variations in precision at individual CEA titres between this assay and the mouse plasma assay described above, there were no discernible qualitative differences.

*Statistics.*—The significance of the analysis of data was calculated using Spearman's test of rank correlation coefficient.

RESULTS

*CEA blood levels related to tumour mass*

CEA rarely became detectable in the blood of mice bearing the human tumours until the total tumour volume reached ~0·6 ml. As individual tumours reached

FIG. 3.—Relation of plasma CEA to tumour volume in colon tumours. Rank correlation HK1; 0·92, $P < 0·01$. HK9; 0·32, $P > 0·05$.

TABLE.—*Correlation of tumour size with blood CEA level (Spearman's rank correlation)*

| Tumour | No. of mice | Tumour volume range (ml) | Tumour mass range (g) | CEA blood levels range (ng/ml) | Correlation: Tumour volume (or mass) *vs* CEA levels | *P* |
|---|---|---|---|---|---|---|
| Colon | | | | | | |
| HK1 | 9 | 0·7–2·2 | | 20–270 | 0·92 | <0·01 |
| HK6 | 20 | 1·1–3 | | 20–250 | 0·26 | NS |
| HK7 | 7 | 1·1–2·3 | | 20–110 | 0·82 | <0·05 |
| HK9 | 25 | 0·8–3 | | 0–220 | 0·32 | NS |
| | 11 | | 0·2–8·1 | 20–640 | 0·66 | <0·05 |
| Breast | | | | | | |
| S32 | 35 | 0·5–5 | | 20–620 | 0·44 | <0·05 |
| Lung | | | | | | |
| p246 | 18 | 0·4–2·4 | | 0–850 | 0·38 | NS |

FIG. 4.—CEA tissue extraction study; multiple implants of HK9 colon.

FIG. 5.—Relationship between tumour mass and plasma CEA levels; multiple implants of HK9 (colon). Rank correlation 0·66, $P < 0{\cdot}05$.

1·5 ml central necrosis set in. Therefore readings were usually taken 4–6 weeks after transplantation, when most of the tumours had grown to 0·5–1·0 ml. Mice bearing tumours of volume < 0·5 ml, or tumours which were frankly necrotic, ulcerated or infected, were discarded.

The results of the bilateral subcutaneous flank volume study are summarized in the Table, and illustrated in Fig. 3. The wide scatter of CEA values within each tumour group was of particular interest, and in only half was there a correlation with tumour volume HK1 (Fig. 3) ($P < 0{\cdot}01$), HK7 ($P < 0{\cdot}05$) and S32 ($P < 0{\cdot}05$).

The greatest discrepancies were found in HK6, where the largest tumours were associated with the lowest plasma CEA levels, whereas in p246 and HK9, mice with relatively small tumour loads occasionally had high levels of circulating CEA. Among HK6, HK9 and p246, it appeared that the highest CEA levels occurred in mice bearing tumour burdens in the mid range. It was considered that some discrepancies might have occurred because of centrilobular necrosis in the larger tumours. However, although postmortem examination confirmed this in certain cases, low CEA levels were found in association with some large tumours in which centrilobular necrosis was not excessive.

The multiple implant study was designed to minimize the effect of centrilobular necrosis. A much wider range (0·5–8 g) could thus also be attained. The results again revealed a wide variation, but an overall linear correlation ($P < 0{\cdot}05$) (Fig. 4). One notable discrepancy was in a mouse carrying one of the largest tumour loads, which had a particularly low plasma level of CEA.

*The relationship of tumour CEA content, tumour mass and CEA blood levels*

HK9 and HK6 tumours were used for this investigation. The HK9 study was an extension of the multiple-implant investigation described above, whereas the HK6 multiple implants were set up specially. (The plasma CEA estimations for this particular group were rendered invalid through technical fault.)

Whereas HK6 demonstrated a clear linear correlation ($P < 0{\cdot}05$) of tumour CEA content with tumour mass, considerable disparity existed in HK9 (Fig. 5) indicating that CEA concentration in xenograft tumours does not remain constant. The relationship of tumour CEA content to plasma levels in HK9 was even more disparate. The possibility had to be considered that where there was wide discrepancy between tumour mass and CEA blood levels, there may have been a corresponding discrepancy in tumour CEA content, but this was clearly not the case.

There is no indication, therefore, that tumours with low or high concentrations were especially associated with corresponding plasma CEA levels.

### DISCUSSION

Previous xenograft investigators have pointed out that tumours must reach a certain size before CEA becomes detectable in the blood (Primus *et al.*, 1973; Mach *et al.*, 1974; Sordat *et al.*, 1974; Miwa *et al.*, 1976; Lewis & Keep, 1981). Indeed, CEA was as rare in mice bearing small tumours in the present study that it became policy not to measure plasma levels until the total tumour bulk was $\sim 1{\cdot}5$ ml. Such a size represents $\sim 5\%$ of the total weight of the tumour-bearing host, which in comparison to malignancies in patients is enormous. In contrast, relatively small tumours in patients are capable of producing very high levels of circulating CEA. This curious observation has also been made for other markers, such as human chorionic gonadotrophin (hCG) (Kameya *et al.*, 1975) and α-foetoprotein (AFP) (Raghavan *et al.*, 1980) produced by xenograft tumours. The reason cannot be wholly explained by assay insensitivity, since small concentrations should be detectable in spite of measurement being inaccurate. It would seem logical that CEA would only become detectable when the amount produced and released by the tumour exceeds that eventually metabolized. However, although it has been shown that CEA is rapidly cleared by the liver in mice (Thomas *et al.*, 1976; Thomas & Hems, 1975) the possibility of a threshold level for CEA clearance has not yet been specifically investigated.

The possibility that there may be a change in CEA synthesis during growth should seriously be considered. Certainly, as tumours grow centrilobular necrosis sets in, and the CEA concentration is naturally smaller when this becomes extensive (Mach *et al.*, 1974). It is not surprising that Lewis & Keep (1981) should find in their single tumour line that CEA concentrations vary widely, because their series produced tumours of vastly differing size. The linear correlation which was found in the HK6 tumours in the present investigation may be because the tumours were harvested early, before serious centrilobular necrosis could have occurred. The possibility that necrosis may be an important factor affecting CEA blood levels has been investigated by the author in a separate study (Quayle, 1982) in which it was found that when necrosis occurred rapidly and extensively, considerable increases in CEA titres were frequent. Nevertheless, the fact that the tumour mass *vs* tumour CEA correlation did not exist in HK9, in spite of similar conditions, indicates that factors other than necrosis are responsible.

A further anomaly identified in this study is the finding that tumour size does not always correlate with plasma CEA. Again this cannot wholly be explained on the basis of tumour necrosis because, if it were, the correlation in the multiple-implant study, which was harvested early, should have been closer than in volume studies.

Until recently, Miwa *et al.* (1976) were the only workers to investigate the relationship between blood CEA and tumour xenograft size. Their claim for a direct correlation does not, however, stand up to close scrutiny, mainly because the number of samples is small (5) and the spread of levels and tumour size uneven. Stragand *et al.* (1980) and Lewis & Keep (1981) failed to demonstrate any correlation in their more recent detailed studies on a single tumour line, but the validity of their observation should perhaps be viewed with some circumspection since they were using tumours which, in some cases, were as large as 8·8 and 7·14 g respectively, which would be expected to contain a considerable degree of central necrosis. Indeed Lewis & Keep (1981) specifically stated that "central necrosis was a consistent feature" in their tumour line. Since the present sudy has indicated behavioural differences between tumour lines, it is conceivable that the two tumour lines used by these authors may not be representative. A further factor, again acknowledged by Lewis & Keep (1981), was that their assay for CEA by a double antibody technique may have been incapable of determining the presence of CEA molecules masked by murine immunoglobulins.

The variable ability of CEA to reflect tumour burden is not wholly unexpected, since a review of the extensive literature indicates that levels of CEA in the blood are affected by numerous other biological variables. Even so, because the validity of xenograft tumour models for such investigations remains in doubt, this failing of CEA may have been exaggerated.

The author acknowledges his gratitude and appreciation to the Breast Unit, Royal Marsden Hospital, London SW3, for financial support; to Professors A. J. S. Davies and A. M. Neville for advice and encouragement; and to Dr M. Ormerod, Miss N. Neylon, Mr M. Capp and Mr K. Gomer for technical assistance.

### REFERENCES

Barrelet, V. & Mach, J-P. (1975) Variation of the CEA level in the plasma of patients with gynaecological cancers during therapy. *Am. J. Obstet. Gynecol.*, **121**, 164.

Borthwick, N. M., Wilson, D. W. & Bell, P. A. (1977) Carcinoembryonic antigen (CEA) in patients with breast cancer. *Eur. J. Cancer*, **13**, 171.

Chu, T. M. & Nemoto, T. (1973) Evaluation of carcinoembryonic antigen in human mammary carcinoma. *J. Natl Cancer Inst.*, **51**, 1119.

Cove, D. H., Woods, K. L., Smith, S. C. H. & 4 others (1979). Tumour markers in breast cancer. *Br. J. Cancer*, **40**, 710.

Dent, P. B. & McCulloch, P. B. (1980) Detection of recurrent metastases using tumour associated serum markers: Validity of results. *Eur. J. Cancer*, **16**, 963.

Dethlefsen, L. A., Prewitt, J. M. S. & Mendelsohn, M. L. (1968) Analysis of tumour growth curves. *J. Natl Cancer Inst.*, **40**, 389.

Di Saia, P. J., Haverback, B. J., Duce, B. J. & Morrow, C. P. (1975) CEA in patients with gynaecological malignancies. *Am. J. Obstet. Gynecol.*, **121**, 159.

Di Saia, P. J., Morrow, C. P. & Haverback, B. J. (1977) CEA in cancer of the female reproductive system: Serial plasma values correlated with disease state. *Cancer*, **39**, 2365.

Gold, P. & Freedman, S. O. (1965) Specific carcinoembryonic antigens of the human digestive system. *J. Exp. Med.*, **122**, 467.

Gropp, C., Havermann, K. & Scheur, A. (1980) The use of carcinoembryonic antigen and peptide hormones to stage and monitor patients with lung cancer. *Int. J. Radiat. Oncol. Biol. Phys.*, **6**, 1047.

Joyce, S., Lobe, T., Cooperman, M. & Martin, E. W. (1980) Direct carcinoembryonic antigen assay in diagnosis and prognosis. *Surgery*, **86**, 627.

Kameya, T., Kuramoto, H. & Suzuki, K. (1975) Properties of human gastric choriocarcinoma cell line (SCH) with two functional markers: Human chorionic gonadotrophin and placental alkaline phosphatase. *Cancer Res.*, **35**, 2025.

Khoo, S. K. & Mackay, E. V. (1976) Carcinoembryonic antigen (CEA) in ovarian cancer: Factors influencing its incidence and changes which occur in response to cytotoxic drugs. *Br. J. Obstet. Gynaecol.*, **83**, 753.

KHOO, S. K., WARNER, N. L., LIE, L-T. & MACKAY, I. R. (1973) CEA activity of tissue extracts: A quantitative study of malignant and benign neoplasms, cirrhotic liver, normal adult and fetal organs. *Int. J. Cancer*, **11**, 681.

KHOO, S. K., WHITAKER, S. U., JONES, I. S. C. & MACKAY, E. V. (1979) Carcinoembryonic antigen in patients with residual ovarian cancer. *Gynecol. Oncol.*, **7**, 288.

LAURENCE, D. J. R., STEVENS, U., BETTELHEIM, R. & 6 others (1972) Evaluation of the role of carcinoembryonic antigen (CEA) in the diagnosis of gastrointestinal, mammary and bronchiol-carcinoma. *Br. Med. J.*, iii, 605.

LEWIS, J. C. M. & KEEP, P. A. (1981) Relationship of serum CEA levels to tumour size and CEA content in nude mice bearing colonic-tumour xenografts. *Br. J. Cancer*, **44**, 381.

MACH, J-P., CARREL, S., MERENDA, C., SORDAT, B. & CEROTTINI, J-C. (1974) *In vivo* localization of radiolabelled antibodies to carcinoembryonic antigen in human colon carcinoma grafted into nude mice. *Nature*, **248**, 704.

MARTIN, E. W., JR, JAMES, K. K., HURTUBISE, P. E., CATALANO, P. & MINTON, J. P. (1977) The use of CEA as an early indicator for gastrointestinal tumour recurrence and second look procedures. *Cancer*, **39**, 440.

MILLER, J. F. A. P., DOAK, S. M. A. & CROSS, A. M. (1963) Role of the thymus in recovery of immune mechanism in the irradiated adult mouse. *Proc. Soc. Exp. Biol. Med.*, **112**, 785.

MIWA, M., SAKURA, H. & KAWACHI, T. (1976) Serum carcinoembryonic antigen level and transplanted colonic tumour size in nude mice. In *Oncodevelopmental Gene Expression* (Ed. Fishman). New York: Academic Press. p. 423.

NOWAK, K., PECKHAM, M. J. & STEEL, G. G. (1978) Variation in the response of xenografts of colorectal carcinoma to chemotherapy. *Br. J. Cancer*, **37**, 576.

ORMEROD, M. G. (1978) Antigenic determinants of carcinoembryonic antigen. *Scand. J. Immunol.*, **8**, 433.

PRIMUS, F. J., WANG, R. H., HANSEN, H. J. & GOLDENBERG, D. M. (1973) Characterization of antibody to carcinoembryonic antigen (CEA) in hamsters xenografted with a human colonic tumour. *Proc. Am. Assoc. Cancer Res.*, **14**, 105.

QUAYLE, J. B. (1982) Tumour lysis as a factor affecting blood levels of CEA *Br. J. Cancer*, **46**, 213.

RAGHAVAN, D., GIBBS, J. & NOGUEIRA COSTA, R. (1980) The interpretation of marker protein assays: A critical appraisal in clinical studies and a xenograft model. *Br. J. Cancer*, **41**, (Suppl. IV), 191.

SORDAT, B., FORTSCHE, R., MACH, J-P., CARRELL, S., OZZELO, L. & CEROTTINI, J-C. (1974) Morphological and functional evaluation of human solid tumours serially transplanted into nude mice. In *Proc. 1st Int. Workshop on Nude Mice* (Eds Rygaard & Povlsen). Stuttgart: Fischer Verlag p. 269.

STEVENS, U., LAURENCE, D. J. R. & ORMEROD, M. G. (1978) Antibodies to lactalbumin interfere with its radioimmunoassay in human plasma. *Clin. Chim. Acta*, **87**, 149.

STEWARD, A. M., NIXON, D., ZAMCHECK, N. & AISENBERG, A. (1974) Carcinoembryonic antigen in breast cancer patients: Serum levels and disease progress. *Cancer*, **33**, 1246.

STRAGAND, J. J., YANG, L-Y. & DREWINKO, B. (1980) Serum CEA levels in a human colonic adenocarcinoma (Lovo) xenograft system. *Cancer Letters*, **10**, 45.

THOMAS, P., BIRBECK, M. S. C. & CARTWRIGHT, P. (1976) A radioautographic study of hepatic uptake of circulating CEA by the mouse. *Biochem. Soc. Trans*, 312.

THOMAS, P. & HEMS, D. A. (1975) The hepatic clearance of circulating native and asialo CEA by the rat. *Biochem. Biophys. Res. Commun.*, **67**, 1205.

VINCENT, R. G., CHU, T. M., FERGEN, T. B. & OSTRANDER, M. (1975) Carcinoembryonic antigen in 228 patients with carcinoma of the lung. *Cancer*, **36**, 2069.

WAALKES, T. P., ABELOFF, M. D., WOO, K. B. & ETTINGER, D. S. (1980) Carcinoembryonic antigen for monitoring patients with small cell carcinoma of lung during treatment. *Cancer Res.*, **40**, 4420.

WANEBO, H. J. (1980) Are carcinoembryonic antigen levels of value in the curative management of colorectal cancer? *Surgery*, **89**, 290.

WARENIUS, H. M. (1979) Identification and separation of mouse and human components of heterotransplanted human tumours. In *Immunodeficient Animals for Cancer Research* (Ed. Sparrow). London: Macmillan. p. 207.

WESTWOOD, J. H. & THOMAS, P. (1975) Studies on the structure and immunological activity of carcinoembryonic antigen: The role of disulphide bonds. *Br. J. Cancer*, **32**, 708.

WESTWOOD, J. H., THOMAS, P., EDWARDS, R. G., SCOPES, P. M. & BARRETT, M. W. (1978) Chemical modifications of the protein of carcinoembryonic antigen: Associated changes in immunological activity and conformation. *Br. J. Cancer*, **37**, 183.